# The clinical relevance of fixation failure after pubic symphysis plating for anterior pelvic ring injuries: an observational cohort study with long-term follow-up

**DOI:** 10.1186/s13037-024-00401-3

**Published:** 2024-05-22

**Authors:** Dmitry Notov, Eva Knorr, Ulrich J.A. Spiegl, Georg Osterhoff, Andreas Höch, Christian Kleber, Philipp Pieroh

**Affiliations:** 1 Department of Orthopedics, Trauma and Plastic Surgery, University of Leipzig, Liebigstrasse 20, 04103 Leipzig, Germany; 2 Clinic for Trauma Surgery and Orthopaedics, Munich Harlaching, Sanatoriumspl. 2, 81545 München, Germany

**Keywords:** Pelvic injury, Symphysis, Implant failure, Functional outcome, Complication

## Abstract

**Background:**

Open reduction and plate fixation is a standard procedure for treating traumatic symphyseal disruptions, but has a high incidence of implant failure. Several studies have attempted to identify predictors for implant failure and discussed its impact on functional outcome presenting conflicting results. Therefore, this study aimed to identify predictors of implant failure and to investigate the impact of implant failure on pain and functional outcome.

**Methods:**

In a single-center, retrospective, observational non-controlled cohort study in a level-1 trauma center from January 1, 2006, to December 31, 2017, 42 patients with a plate fixation of a traumatic symphyseal disruption aged ≥ 18 years with a minimum follow-up of 12 months were included. The following parameters were examined in terms of effect on occurrence of implant failure: age, body mass index (BMI), injury severity score (ISS), polytrauma, time to definitive treatment, postoperative weight-bearing, the occurrence of a surgical site infection, fracture severity, type of posterior injury, anterior and posterior fixation. A total of 25/42 patients consented to attend the follow- up examination, where pain was assessed using the Numerical Rating Scale and functional outcome using the Majeed Pelvic Score.

**Results:**

Sixteen patients had an anterior implant failure (16/42; 37%). None of the parameters studied were predictive for implant failure. The median follow-up time was six years and 8/25 patients had implant failure. There was no difference in the Numerical Rating Scale, but the work-adjusted Majeed Pelvic Score showed a better outcome for patients with implant failure.

**Conclusion:**

implant failure after symphyseal disruptions is not predictable, but appears to be clinically irrelevant. Therefore, an additional sacroiliac screw to prevent implant failure should be critically discussed and plate removal should be avoided in asymptomatic patients.

## Introduction

Traumatic symphyseal disruptions are rare, potentially life-threatening injuries, that are usually stabilized with plate fixation [1–3]. However, plate fixation shows a high incidence (12–75%) of implant failure [4–7]. Different implant designs and surgical techniques have been developed to minimize the risk for this [2, 8–11]. Alternatively, additional sacroiliac (SI) screw fixation, a primary symphyseodesis or elective plate removal have been discussed to reduce implant failure [12–16].

Currently, possible predictors of implant failure, such as anterior plate type [6, 17, 18], posterior stabilization [12, 18], fracture classification [5, 6] or demographics [15, 19] are controversially discussed. Furthermore, the clinical relevance of implant failure remains unclear due to conflicting reports and a low revision rate [6, 15, 17, 20].

The aim of the present study was to identify potential predictors of implant failure following plate fixation of traumatic symphyseal disruptions. Secondarily, the impact of implant failure on functional outcome and pain was investigated.

## Methods

### Study design

**Fig. 1:**
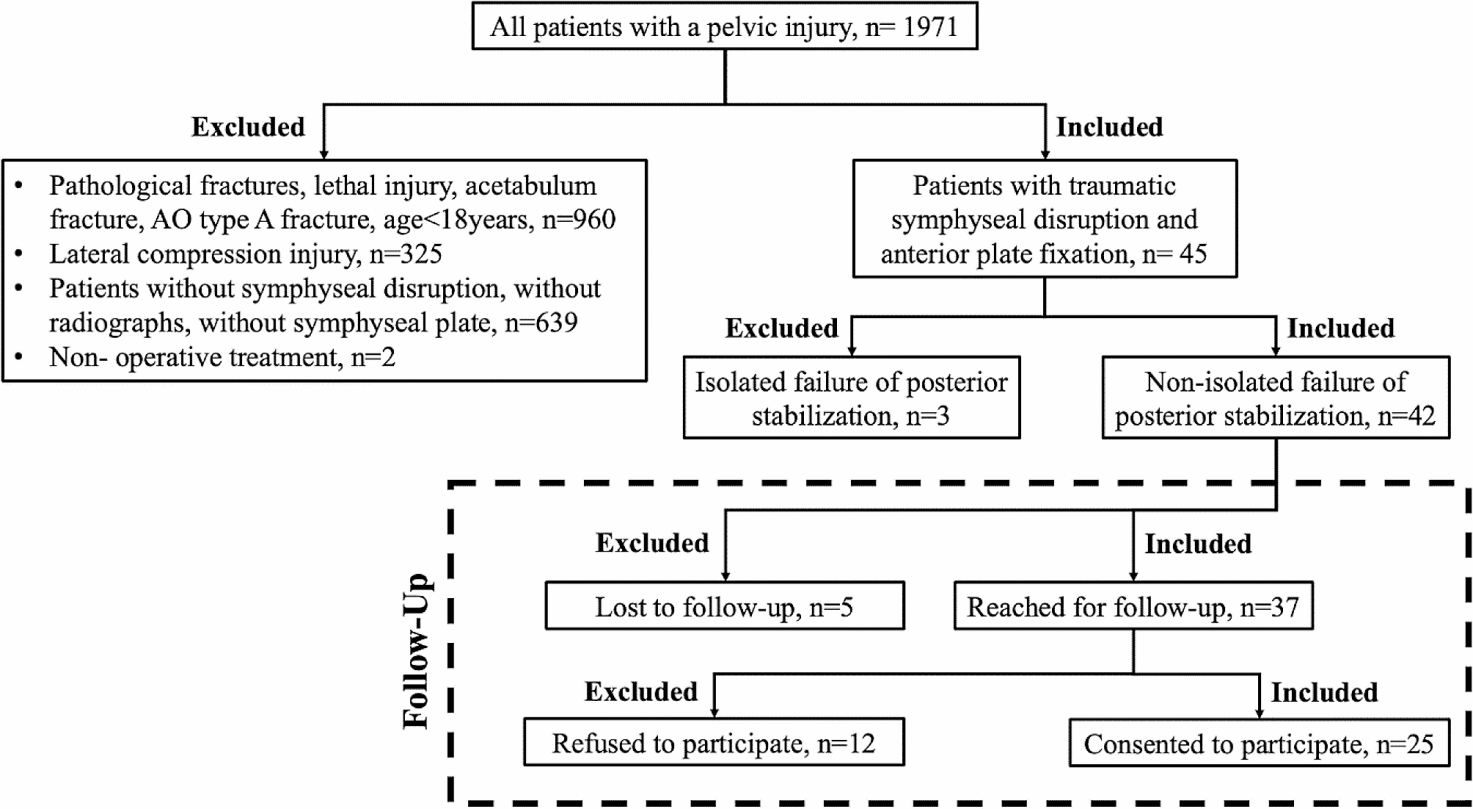
Patient selection flow-chart

A single-center, retrospective, observational non-controlled cohort study was performed in a level-1 trauma center. All patients were consecutively enrolled and included if they were treated with a plate fixation of a traumatic symphyseal disruption between January 1, 2006, and December 31, 2017, were ≥ 18 years of age, and had a minimum follow-up of 12 months. Patients with pathological fracture, a lethal injury, acetabulum fracture, AO type A fracture, Young and Burgess lateral compression injury or posterior implant failure were excluded. Of the 42 patients identified, 37 patients could be reached by telephone, 25 patients consented to participate in the study. Patient selection is shown in Fig. 1.

### Outcome measures

Implant failure was defined according to the criteria published by Collinge et al. (interval backout, lysis halo around the screw threads, breakage of plate or screws or separation between screw head and plate) [20]. If implant failure occurred more than once in a patient, the implant failure was counted only once.

The following parameters influencing the occurrence of implant failure were evaluated: age, body mass index (BMI), injury severity score (ISS), presence of polytrauma, time to definitive treatment, postoperative weight bearing, occurrence of a surgical site infection. Fracture severity was also analyzed. All pelvic injuries were classified according to the AO classification of 2018 and Young and Burgess classification. For analyses regarding the impact of the posterior injury, sacral fractures were compared to injuries of sacroiliac joint. If patients had a sacral fracture and a sacroiliac joint injury, they were classified as having a sacroiliac joint injuries. To assess surgical predictors of implant failure, the type of anterior fixation, plate type and type of posterior stabilization was examined.

The impact of implant failure on pain was assessed using the Numerical Rating Scale and functional outcome was investigated using Majeed Pelvic Score. Since 3 patients did not have a regular job at the time of injury, additionally the relative Majeed Pelvic Score was assessed in order to compare all patients. It was defined as the percentage of the maximum score that could be achieved.

### Statistical analysis

The data processing and statistical analysis was carried out using IBM SPSS Statistics 27® (IBM Corporation Armonk, NY, USA) and Microsoft Office Excel 2021® (Microsoft Corporation, Redmond, WA, USA). Mean ± standard deviation was given for Gaussian distributed data. For non- Gaussian distributed data, median [interquartile range (IQR)_25%_; IQR_75%_] was given. Group comparisons of nominal data were carried out using crosstabs and chi-square tests. Gaussian distributed data were analyzed using the t-test and non-Gaussian distributed data by the Wilcoxon / Mann Whitney U test. The level of statistical significance was defined at a p- value < 0.05.

## Results

A total of 47 patients with a traumatic symphyseal disruption aged ≥ 18 years were identified. Of these, two were excluded due to non-operative treatment and three related to solely posterior implant failure. Thus, a total of 42 patients were included and analyzed.

The included patients (39 male, three female) were 49.3 ± 16 years old, had a BMI of 27.5 ± 3.7 kg/m², an ISS of 21.2 ± 9.9 points and a median time to definite operative treatment of 2 [0.8;4] days. A total of 28/42 patients (67%) had polytrauma (ISS > 16).

Implant failure was observed in 16/42 cases (38%). It occurred in median after 72.5 [24.3;183] days and in 5/16 during initial hospitalization. Implant failure occurred in 4/16 patients during the first 30 days after surgery. Implant failure occurred twice in 2/16 patients. According to the criteria of Collinge et al., screw loosening occurred in 13/16 patients, screw breakage in 2/16 and plate breakage in 1/16.

### The influence of patient’s demographics, fracture severity and posterior injury

Age, BMI, ISS, polytrauma, time to surgery (TTS), BMI, post-operative weightbearing (WB) and surgical site infections (SSI) were not associated with implant failure (*p* > 0.05; Table 1).

**Table 1:** Comparison of patient’s demographics between patients with and without implant failure showed no significant differences (*p* > 0.05). Data are given as mean ± standard deviation (SD) (median), median (interquartile range [IQR]_25%_; IQR_75%_) or number (n). ISS injury Severity Score, BMI body mass index, TTS time from admission till definite surgery in days, WB weight bearing, f full, partial p, w wheelchair, b bedridden, SSI post-operative wound infection

	Implant failure (*n* = 16)	No failure (*n* = 26)	*p*-Value
Age [years]	53.3 ± 15.3	46.3 ± 16.3	*p* = 0.21
BMI [kg/m²]	28.6 ± 3.1	26.8 ± 3.7	*p* = 0.14
ISS	21.4 ± 11.3	21.1 ± 9.2	*p* = 0.93
Polytrauma (n=) yes: no	5:11	8:18	*p* > 0.99
TTS [days]	1.5 (0;4)	2 (1;4)	*p* = 0.99
WB (n=) f: p:w: b	5:10:0:1	13:5:7:1	*p* = 0.34
SSI (n=) yes: no	4:12	5:21	*p* = 0.71

The distribution of fractures is shown in Table 2. When comparing type B and C fractures, there were no significant differences regarding implant failure in either anterior-only (*p* = 0.18) or anterior-posterior (*p* = 0.20) treated patients. When comparing anterior-posterior compression injuries (APC) II to > APC II injuries, there were no differences. There was no difference in APC II vs. > APCII, for either anterior-only (*p* > 0.99) or anterior- posterior (*p* = 0.55) treated patients.

The presence of implant failure did not differ between patients with sacral fractures (*n* = 9/42; implant failure *n* = 1/16) and sacroiliac injuries (*n* = 26/42; implant failure *n* = 15/16; *p* = 0.12).

**Table 2:** Fracture distribution according to the AO and Young and Burgess classification for all patients and splitted regarding implant failure. Patients were further subdivided regarding the presence (+ PF) or absence (-PF) of a posterior fixation. The type of posterior fixation is presented: sacroiliac screws (SIS), SIS combined with a spinopelvic fixation (TSPF). Patients treated with an iliac plate and SIS are marked by *. One case treated with an iliac plate is not included in the table (AO C1.3, VS). APC anterior posterior compression, VS vertical shear, CM combined mechanism

	Subtype	all	implant failure		no implant failure	
*PF*		*n-PF/n + PF*	*n-PF/n + PF*	*Type PF (SIS/TSPF)*	*n-PF/n + PF*	*Type PF (SIS/TSPF)*
**AO classification**	**B**	**23/4**	**8/3**	**3/0**	**13/1**	**1/0**
	1.2	2/0	0/0		2/0	
	2.3	17/4	6/3	3/0	11/1	1/0
	3.1	2/0	0/0		2/0	
	3.3	2/0	2/0		0/0	
	**C**	**2/13**	**2/3**	1*/1	**0/10**	2*/9
	1.2	2/2	2/0		0/2	1*/1
	1.3	0/9	0/3	1*/1	0/6	1*/5
	2.3	0/2	0/0		0/2	0/2
**Young and Burgess classification**	APC I	4/1	0/1	1/0	4/0	
	APC II	17/4	8/2	2/0	9/2	1/1
	APC III	6/3	3/1	0/1	2/4	1*/3
	VS	0/4	0/2	1/0	0/2	0/2
	CM	0/3	0/0		0/3	1/2

### Surgery-related predictors

A total of 40/42 patients were treated with a single anterior plate, and 2/42 with a double plate (1/2 with implant failure). In 3/42 patients, reconstruction plates with 3.5 mm screws were used for anterior stabilization. The remaining 39/42 patients were treated with a dynamic compression plate of 4.5 mm. Of the patients with a single plate, a four-hole plate was used in 36/40 cases (22/36 without and 14/36 with implant failure). The remaining four patients were treated with a five-, six- ten- or 12-hole plate. Of these only the patient treated with the five-hole plate had an implant failure. The plate choice does not influence the occurrence of implant failure (*p* > 0.99). Of the patients with implant failure, 3/16 (7.14%) required revision surgery, each one was treated by double plate fixation, a longer plate with spinopelvic fixation, and single plate exchange.

Additional posterior stabilization was performed in 17/42 patients (40.5%) (Table 2). Posterior stabilization was either performed with SI screws (5/17), SI screws combined with a spinopelvic fixation (9/17), SI screws combined with an iliac plate (2/17) or with an iliac plate (1/17). There was no difference in the incidence of implant failure when comparing SI screws with spinopelvic fixation (*p* = 0.46).

### Pain and functional outcome

The median time to follow-up was 6 (2.5; 7.5) years. Of 25 patients, eight had implant failure. The Numerical Rating Scale was 4 ± 2.36 and not significantly different between patients with (3.38 ± 2.01) and without (4.29 ± 2.49) implant failure (*p* = 0.29). The answers to the Majeed Pelvic Score are shown in Table 3.

**Table 3:** Distributions of answers (n) given to the categories of the Majeed Pelvic Score for all (A), patients with implant failure (IF) and patients without implant failure (NIF). The answers are followed by the number of points in brackets assigned to the answer

Category	Answer	A/IF/NIF
**Pain**	Tolerable, but limits activity (15)	3/0/1
	With moderate activity, abolished by rest (20)	1/0/1
	Mild, intermittent, normal activity (25)	8/3/5
	Slight, occasional or no pain (30)	13/5/8
**Work**	No regular work (0–4)	6/0/6
	Change of job (12)	2/1/1
	Same job, reduced performance (16)	2/1/1
	Same job, same perormance (20)	12/4/8
**Sitting**	Painful if prolonged or awkward (6)	8/1/7
	Uncomfortable (8)	1/0/1
	Free (10)	16/7/9
**Sexual intercourse**	Painful (0–1)	4/0/4
	Painful if prolonged or awkward (2)	1/1/0
	Uncomfortable (3)	2/0/2
	Free (4)	18/7/11
**Standing**	Wheelchair (4)	1/0/1
	Two sticks (8)	1/0/1
	No sticks (12)	23/8/15
**Gait unaided**	Cannot walk or almost (0–2)	1/0/1
	Moderate limp (8)	1/0/1
	Slight limp (10)	8/3/5
	Normal (12)	15/5/10
**Walking distance**	Bedridden or few metres (0–2)	1/0/1
	Very limited time and distance (4)	3/0/3
	One hour without sticks slight pain or limp (10)	4/2/2
	Normal for age and general condition (12)	17/6/11

Grading the Majeed Pelvic Score yielded the following distribution: excellent *n* = 17/25 (implant failure *n* = 8/8, no implant failure *n* = 9/17), good *n* = 3/25 (no implant failure *n* = 3/17), fair *n* = 2/25 (no implant failure *n* = 2/17), poor *n* = 3/25 (no implant failure *n* = 3/17). The Majeed Pelvic Score was 82.8 ± 18.39 for all patients, 90.13 ± 8.37 for patients with implant failure and 79.35 ± 20.91 for patients without implant failure. There was no significant difference (*p* = 0.177) between patients with and without implant failure.

Three patients (two with implant failure) had no regular work before their pelvic injury. The relative Majeed Pelvic Score was 84.77%±17.86% for all patients, 95%±4.14% for patients with implant failure and 79.96%±19.85% for patients without implant failure, revealing a better outcome for patients with implant failure (*p* = 0.047). Analyzing the categories of the Majeed Pelvic Score by comparing the most favorable outcome to the remaining answers, presented no significant differences in any category between patients with and without implant failure(*p* > 0.05).

## Discussion

No significant associations between patient characteristics (e.g. age, BMI, ISS) or treatment-specific factors (e.g. time to surgery, post-operative weight-bearing protocol) and the occurrence of implant failure was observed. Factors, such as fracture severity, additional posterior stabilization, and the specific type of posterior injury did not influence implant failure rates. The Majeed Pelvic Score was higher in the implant failure group after adjusting it to the previous work of patients. The implant failure rate of 37% is comparable to previous reports [12, 15, 18, 19]. Furthermore, the median time to implant failure of approximately 10 weeks is comparable to Rojas et al. (seven weeks), Eastman et al. (13 weeks) and Avilucea (16 weeks) et al. but earlier than reported by Morris et al. (one year) [5–7, 12].

The inability to predict implant failure was previously reported in a more heterogeneous group of pelvic ring injuries [21]. As in the study of Frietman et al. no predictors of implant failure in patients’ demographics could be detected [15]. Tseng et al. reported, that males suffer more often from implant failure [19]. Due to the gender inhomogeneity of the cohort presented here, with 93% male patients, this finding could either be proven or disproven.

Conflicting reports exist, regarding the effect of fracture severity according to the AO classification, and it is poorly documented for the Young and Burgess classification [5, 6, 15, 20]. The advantage of our study is the use of both the Young and Burgess and AO classification, particularly because of the conflicting recommendations for comparable injuries associated with the use of different classification systems. Performing a global survey yielded a predominant use of stand-alone anterior plating especially in Europe for AO type B1.1 injuries [22]. In contrast, a survey in the UK revealed a favored treatment using an anterior plate with an additional SI screw for APC II injuries [10]. Different recommendations may result from to a more heterogeneous injury pattern and displacement within similar classified injuries as known from lateral compression fractures [23]. This hypothesis is supported by the recommendation of Gill et al. performing an individual assessment of stability and required stabilization even in similarly classified injuries [10]. The fracture classification was not predictive of implant failure in the present study.

While the choice of a two- vs. a four-hole plate affects the occurrence of implant failure [17], the choice of longer plates or double plating does not affect implant failure [6, 15, 18, 19].

Besides fracture classification, the type of posterior injury may affect implant failure. Eastman et al. determined implant failure predominantly in patients suffering from sacroiliac joint injuries [7]. This may be due to the underestimation of instability or micro-instability caused by these injuries, or the lack of ability to detect them on static imaging [7, 18]. Such instabilities could be addressed with an additional posterior fixation resulting in a reduction of implant failure [12]. However, the present study as well as previous studies were unable to support these finding [5, 6, 15, 18, 19].

In addition to different classifications, different weight bearing recommendations for the same injury pattern can affect implant failure [10]. The present study could not support this thesis, which can be explained by a possible incompliance of the patients with partial weight bearing which could not be excluded [7].

The impact of implant failure on functional outcome is still a matter of debate [15, 17]. Frietman et al. supported the view, that implant failure could be the result of healing and the return of mobility within the pelvic ring and therefore should not be considered as a complication [15]. Pain levels did not differ in this study comparable to previous reports [17].

Compared to previous studies, the Majeed Pelvic Score was higher in the present study [15, 18, 19, 24, 25]. However, there are differing opinions on the impact of implant failure on the functional outcome as followed: no impact [19, 26], a tendency for better outcome without significance for intact implants [5, 17] or implant failure [15]. In the present study, the implant failure group showed a significantly better outcome adjusting the Majeed Pelvic Score to the work category.

The present study was limited by the retrospective design, the predominance of male patients, and the small number of patients, which reduced the power. Functional outcome could be estimated in only 60% (25/42) of the cohort.

In conclusion, implant failure is a common radiologic phenomenon with little or no relevance to revision indication or functional outcome [20]. In particular, screw loosening should not be overemphasized and, as previously suggested, radiologic analysis may not necessarily predict functional outcome [15, 27]. Therefore, plate removal in asymptomatic patients is not recommended and the addition of a sacroiliac screw should be critically discussed.

## Conclusion

Anterior implant failure after symphyseal disruption is common and there are currently no factors that predict the occurrence of implant failure. Of note, the group without implant failure is not superior to patients with implant failure in terms of functional outcome, challenging the general recommendation of additional sacroiliac screws to prevent implant failure and the consideration of plate removal in asymptomatic patients.

## Data Availability

Data that supports the findings of this study is available on reasonable request from the senior author.
